# Quantifying lipofuscin in retinal pigment epithelium in vivo by visible-light optical coherence tomography-based multimodal imaging

**DOI:** 10.1038/s41598-020-59951-y

**Published:** 2020-02-19

**Authors:** Zahra Nafar, Rong Wen, Ziqiang Guan, Yiwen Li, Shuliang Jiao

**Affiliations:** 1 Department of Biomedical Engineering, Florida International University, 10555 W Flagler St, Miami, FL 33174 USA; 2 Bascom Palmer Eye Institute, University of Miami Miller School of Medicine, 1638 NW 10 Ave, Miami, FL 33136 USA; 3 Department of Biochemistry, Duke University School of Medicine, 307 Research Dr, Durham, NC 27710 USA

**Keywords:** Imaging, Diagnostic markers, Diagnostic markers

## Abstract

Lipofuscin in the retinal pigment epithelium (RPE) is the major source of fundus autofluorescence (FAF). A technical challenge to accurately quantify the FAF intensities, thus the lipofuscin concentration, is to compensate the light attenuation of RPE melanin. We developed the VIS-OCT-FAF technology to accomplish optical coherence tomography (OCT) and FAF simultaneously with a single broadband visible light source. We demonstrated that light attenuation by RPE melanin can be assessed and corrected using the depth-resolved OCT signals. FAF images from albino and pigmented rats showed that without compensation, FAF signals from pigmented rats are lower than that from albinos. After compensation, however, FAF signals from pigmented rats are higher. This finding is supported by measurements of lipofuscin fluorophore A2E in the RPE using liquid chromatography/mass spectrometry (LC/MS) showing that compensated FAF intensities correlate linearly with A2E contents. The present work represents an important step toward accurately assessing RPE lipofuscin concentrations by FAF.

## Introduction

Lipofuscin in the retinal pigment epithelium (RPE) cells, upon excitation with light in its absorption spectrum (~488 nm in clinical systems), emits fluorescence known as the fundus autofluorescence (FAF)^1–3^. FAF is thus a measure of lipofuscin concentration in the RPE, a monolayer of pigmented cells located next to photoreceptors in the retina with multiple functions^4,5^. Rod photoreceptors renew their entire outer segments (OS) every 10 days by adding new discs to the OS base and shedding OS tips, which are phagocytosed and digested by the RPE cells^6,7^. The daily phagocytosis of OS tips leads to the accumulation of the nondegradable end product lipofuscin, a complex lipid/protein aggregate^8,9^. It has been suggested that RPE lipofuscin plays a detrimental role in degenerative retinal diseases including age-related macular degeneration (AMD) and Stargardt disease^8,10^.

The first identified fluorophore in RPE lipofuscin is a pyridinium bisretinoid A2E (N-retinyl-N-retinylidene ethanolamine), a byproduct of the photoreceptor visual cycle and a major fluorophore of lipofuscin^11–13^. A2E has been suspected to affect RPE functions, and therapeutic strategies targeting A2E are being developed for AMD and Stargardt disease^14–16^.

FAF carries information of lipofuscin in the RPE cells. It is known that FAF intensities increase with age^17^, which reflects the accumulation of lipofuscin in the RPE with age^18,19^. FAF intensities thus can be regarded as a biomarker of RPE cell aging. Local and global alteration of FAF intensities has been seen in retinal degenerative diseases, such as AMD and Stargardt disease^20^. The clinical usefulness of FAF imaging is limited, however, as the currently available FAF technologies are not capable of measuring the true FAF intensities accurately. For example, it is difficult to use FAF for diagnosis and disease monitoring, since comparison of FAF images taken from different individuals or from the same individual taken at a different time requires quantitative measurement of the true FAF intensities. Technologies capable of quantitatively measuring the true FAF intensities could overcome this limitation and expand the clinical application of FAF^17,21–27^.

Quantitatively measuring the true FAF intensities faces two major technical challenges. The first one is to standardize the intensities of a FAF image. Delori and co-workers^17,21,28^ made a significant contribution in addressing this issue by placing a reference fluorescent target of known fluorescence efficiency in the intermediate retinal imaging plane. The fluorescence efficiency ξ is defined as the product of the fluorophore concentration (C), the molecular quantum yield (Q), the extinction coefficient (ε), and the thickness of the target (d), ξ = C × Q × ε × d. The reference target blocks a small portion of the field of view and is imaged each time a FAF image is taken. Thus it is possible to standardize the FAF intensities using the fluorescence intensity of the reference target.

The second challenge is to compensate the signal attenuations caused by media anterior to the RPE (pre-RPE media) and melanin in the RPE. The pre-RPE media are the ocular tissues anterior to the RPE, including the cornea, the aqueous humor, the lens, the vitreous, and the neuronal retina. The pre-RPE media and RPE melanin attenuate both the excitation light before it reaches the RPE, and the emitted FAF signals on their way from the RPE to the detector. These attenuations cannot be measured directly^29,30^, making compensation more difficult.

We reasoned that the depth-resolved OCT signals from the RPE could be used as a reference for signal attenuation when the OCT and FAF images are generated simultaneously from the same photons. To test this idea, we developed the VIS-OCT-FAF technology, a visible-light optical coherence tomography (VIS-OCT) based multimodal imaging technology by using a single broadband light source to generate OCT and FAF images simultaneously. We have demonstrated in our previous studies that VIS-OCT-FAF can compensate for the attenuation caused by the pre-RPE media^31,32^. In addition, by using a pair of fluorescent reference targets with known fluorescence efficiency and reflection coefficient inserted in the intermediate retinal imaging plane, it can also eliminate the factors like power fluctuations of the light source and gain variations of the detector^31,33^.

Here we report that signal attenuation by RPE melanin can be effectively compensated with the VIS-OCT-FAF technology using the simultaneously acquired OCT signals from the RPE as a reference. The present work is a significant step toward measuring the true FAF intensities *in vivo*, which could expand the clinical application of FAF to monitor retinal diseases, including disease progression and treatment outcomes.

## Results

### System performance

**Figure 1:**
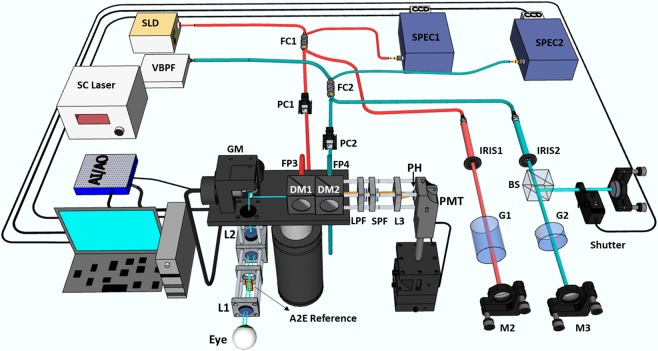
A schematic of the VIS-OCT-FAF imaging system. The system integrates two OCTs, a VIS-OCT (blue) and a NIR-OCT (red), and a confocal FAF detection module. In the sample arms of the two OCTs, the VIS and NIR light is combined by two dichroic mirrors (DM1 and DM2), scanned together by the X-Y galvanometer scanning mirrors (GM), and delivered to the retina by a pair of lenses (L1 and L2). The corresponding OCT signals are detected by two spectrometers (SPEC1 and SPEC2). The FAF signal is detected by a photomultiplier tube (PMT) through a set of filters and a pinhole (PH). SLD: Superluminescent Diode; SC: Supercontinuum Laser; VBPF: Variable band-pass filter; M1-3: Reference arm mirrors; IRIS1-2: iris; G1-2: BK7 glass plates; BS: beam splitter; FC1-2: 2 × 2 fiber coupler; FP1-4: fiber collimator; PC1-2: polarization controller; L1-3: lens; LPF: long-pass filter; SPF: short-pass filter.

We built a VIS-OCT-FAF system (Fig. 1) for the study. The VIS-OCT-FAF system integrated a spectral-domain VIS-OCT, a spectral-domain near-infrared (NIR) OCT, and a confocal FAF detection channel on a single platform. The system used a supercontinuum light source for both VIS-OCT and FAF excitation. The filtered output of the light source had a center wavelength of 480 nm and a bandwidth of 30 nm. The axial resolution and sensitivity at a path-length difference of 0.5 mm of the VIS-OCT were measured to be 6 μm and 85 dB, respectively. The signal roll-off at a depth of 2 mm was measured to be −8 dB, which was compensated in quantification of the FAF intensities. The system acquires spatially registered VIS-OCT and FAF images simultaneously at a speed of 24k lines per second, determined by the line rate of the CCD camera of the OCT spectrometer. The NIR-OCT was used for alignment and identifying the retinal area of interest (AOI). Upon activation of data acquisition, the visible light is turned on and scanned across the AOI. The VIS-OCT and FAF images were acquired simultaneously and streamed to a computer.

### Characterization of the reference target

We fabricated a reference target for both fluorescence and reflection, which was placed in the intermediate retinal imaging plane, similar to the configuration used by Delori and co-workers^34–36^. The target was a piece of poly methyl methacrylate (PMMA) containing synthesized A2E as the fluorescent dye. It was made by dissolving PMMA and A2E in an anisole solvent (24 mg of A2E in 20 ml of 4% PMMA in anisole). The solution was cured by heat in an aluminum mold and the target was cut to the desired shape with a CO_2_ laser. The reflectance of the reference target was measured to be 0.04, consistent with the theoretical specular reflectance of PMMA with a refractive index of 1.49. The emission spectrum of the reference target was identical to that of synthesized A2E in methanol with peak emission at 562 nm when measured with a custom-made spectrofluorometer at an excitation wavelength of 488 nm. The quantum yield of A2E was measured to be 0.003 ± 0.001 at an excitation of 488 nm by using fluorescein and rhodamine solutions as references, consistent with published data^37^.

### *In vivo* VIS-OCT-FAF imaging

**Figure 2:**
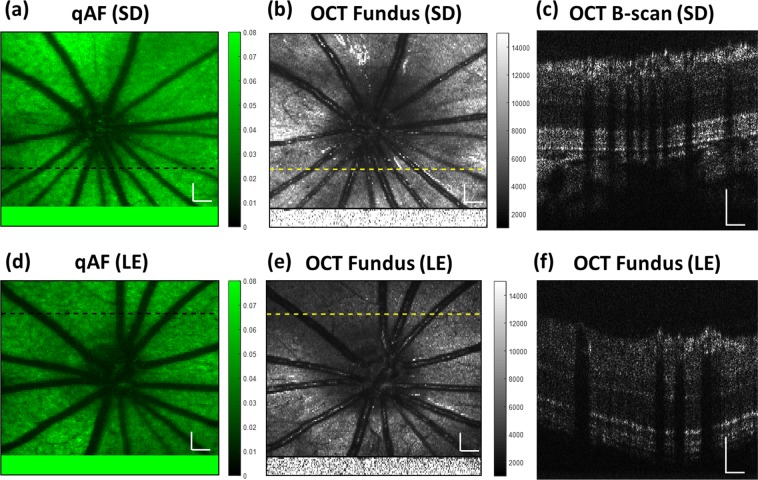
Representative FAF and OCT images from albino (SD) and pigmented (LE) rats. The FAF and OCT images were acquired simultaneously. The intensities of the FAF (**a**,**d**) were normalized to the fluorescence intensity of A2E in the reference target at the bottom of each image (**a**,**d**) (see text for details). The OCT fundus images (**b**,**e**) are an en face view of the 3D OCT data projected onto the X-Y plane. The reflectance image of the reference target is shown at the bottom of each 3D OCT image (**b**,**e**). B-Scan OCT images in pannels c and f were taken at the location marked with the dashed lines, respectively. The normalized FAF intensities from the albino rat (**a**) appear brighter than that of the pigmented rat (**d**). Bars: 200 µm.

We imaged 11-week old albino Sprague Dawley rats (SD, 3 animals, 6 eyes) and pigmented Long Evans rats (LE, 3 animals, 6 eyes) using the VIS-OCT-FAF system to examine the effect of attenuation compensation. Figure 2 shows representative quantified FAF (qAF) and VIS-OCT images. Each qAF image was obtained by normalizing the intensities of each pixel of the raw FAF image, *I*_*FAF*_, to the fluorescence intensity of the reference target, *I*_*ref-AF*_ (*qAF* = *I*_*FAF*_/*I*_*ref-AF*_, Eq. 3 in Methods). The fluorescence image of the reference target is shown at the bottom of each qAF image (Fig. 2a,d). The qAF image from an albino rat (Fig. 2a) appears brighter than the qAF image of the pigmented rat (Fig. 2d).

The *en face* view of the 3D OCT image (projection of the data onto the XY plane) of the albino and pigmented rats is shown in Fig. 2b,e. The reflectance image of the reference target is shown at the bottom of Fig. 2b,e. Cross-sectional OCT images of the albino and pigmented rats are presented in Fig. 2c,f, respectively.

### Compensation of signal attenuation by RPE melanin

**Figure 3:**
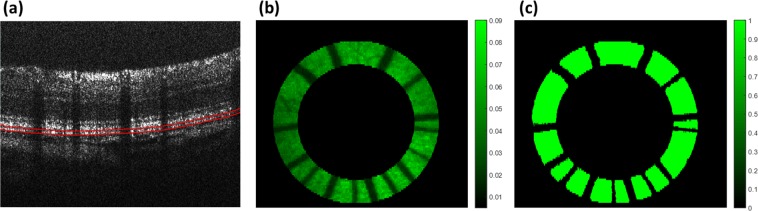
Image processing for data analysis. (**a**) The OCT signals from the RPE were manually segmented on the cross-sectional images between the two red lines. (**b**) A ring area on the fundus projection was selected to remove the optic disc and surrounding area for calculating the averaged qAF and qOCT. (**c**) The mask generated by binarization to remove blood vessel shadows.

We obtained the OCT signals of the RPE, *I*_*RPE-OCT*_, by manually segmenting the RPE layer on each cross-sectional image (B-scan) and summing the signals between the two segmented boundaries (Fig. 3a, red lines) that contain the RPE layer. The quantified OCT signals of the RPE (qOCT) were obtained by normalizing the raw OCT intensities to the OCT signal from the reference target *I*_*ref-OCT*_: *qOCT* = *I*_*RPE-OCT*_*/I*_*ref-OCT*_.

**Figure 4:**
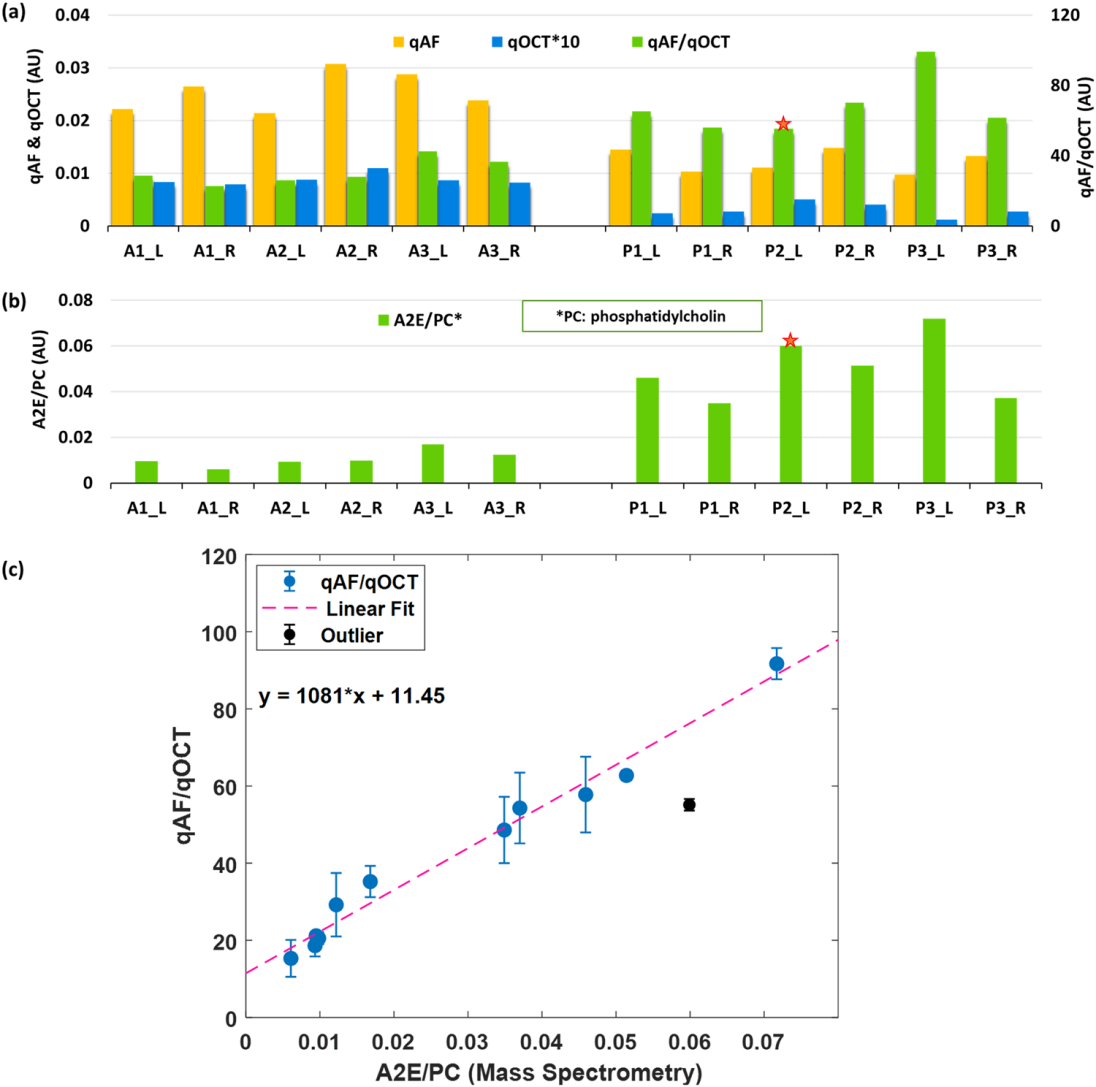
Correlation between FAF intensities and A2E amounts. Data presented are from 6 eyes of 3 albino rats (A1, A2, and A3; L: left eye; R: right eye), and 3 pigmented rats (P1, P2, and P3; L: left eye; R: right eye). The FAF intensities qAF (**a**, yellow bars) from the eyes of SD rats are higher than that of LE rats, so are the intensities of qOCT (**a**, blue bars). When the qAF is normalized to qOCT, the qAF/qOCT is actually higher in the pigmented LE rats than in the albino rats (a, green bars), which are in good agreement with the A2E amounts directly measured by LC/MS (**b**). There is a linear correlation between qAF/qOCT (mean ±  Std) and A2E (R2 = 0.98) (**c**). The qOCT values presented are multiplied by 10 (qOCT × 10) due to their low levels. *: Outlier; AU: arbitrary unit.

**Figure 5:**
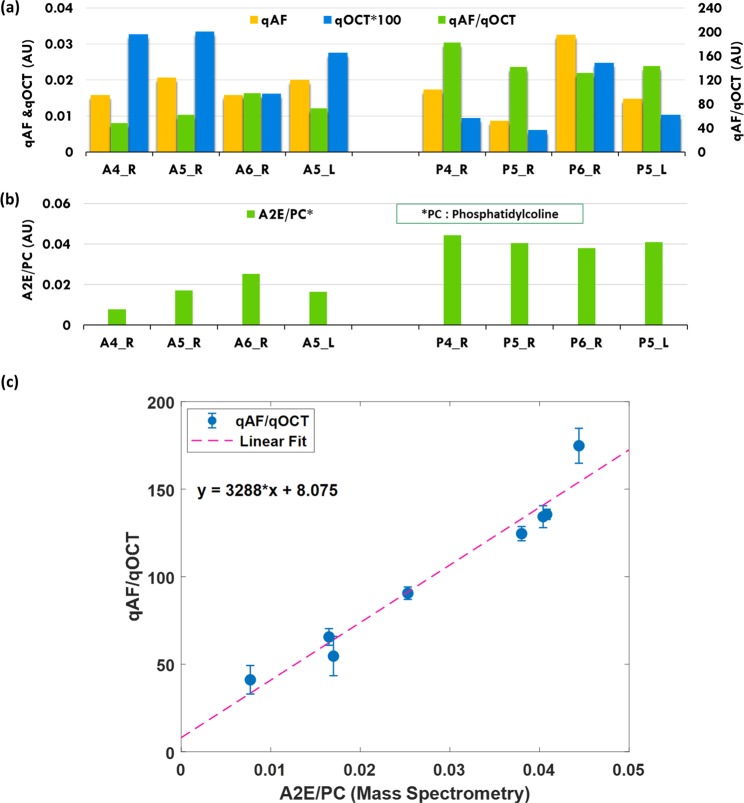
Correlation between FAF intensities and A2E amounts with a DAPI reference target. Experiments were similar to those presented in Fig. 4, except that the reference target was a commercially available PMMA slide with DAPI fluorescent dye. Similar to the results presented in Fig. 4, the qAF and qOCT of SD rats are higher than that from LE rats (**a**, blue and yellow bars, respectively), whereas the compensated FAF intensities qAF/qOCT are higher in the pigmented LE rats (**a**, green bars). The A2E amounts, directly measured by LC/MS, are again in good agreement with qAF/qOCT (**b**). A linear correlation exists between qAF/qOCT (mean ±  Std) and A2E amount (R^2^ = 0.95) (**c**).

To calculate the mean of qAF and qOCT for each eye, a ring area centered at the optic disc was selected. It had an inner diameter of 1.38 mm and an outer diameter of 1.95 mm (Fig. 3b,c). The blood vessel areas are excluded from the calculation (Fig. 3b,c). The qAF, qOCT, and qAF/qOCT were calculated from this area in each of the eyes presented in Figs. 4 and 5.

The intensities of qAF and qOCT calculated from each of the 6 eyes of the 3 albino rats and 6 eyes of the 3 pigmented rats are presented in Fig. 4. The qAF intensities from the albino animals are higher than that from the pigmented rats (Fig. 4a, yellow bars). The qOCT intensities of the albino rats are also higher than that of the pigmented rats (Fig. 4a, blue bars), apparently due to light absorption of melanin in the RPE. When qAF was normalized to qOCT, however, the compensated FAF intensities qAF/qOCT of the pigmented animals are higher than that of the albino rats (Fig. 4a, green bars). The averaged qAF/qOCT of pigmented rats (67.79 ± 16.29, mean ± standard deviation or SD, n = 6) is more than twice of that from the albino rats (30.65 ± 7.14, mean ± SD, n = 6, P < 0.0005, Student’s t-test). The dramatic jump in readings from qAF to qAF/qOCT of the pigmented rats demonstrates significant signal attenuation caused by RPE melanin and the compensation effect by normalizing qAF to qOCT.

The higher qAF/qOCT in pigmented rats should correspond to higher concentrations of lipofuscin fluorophores in the RPE. To confirm this, we measured the A2E content of the same eyes by LC/MS. Eyes were collected immediately after imaging. The RPE-choroid preparation was dissected from each eye and the total lipids were extracted. The measured A2E content by LC/MS in each sample was normalized to the amount of phosphatidylcholine 34:1 (PC 34:1), a major phospholipid of the retina^38^. As shown in Fig. 4b, A2E contents in the pigmented rats are indeed higher than that in the age-matched albino rats, in good agreement with the calculated qAF/qOCT. A linear relationship was revealed between qAF/qOCT and the A2E contents measured by LC/MS (R^2^ = 0.98, Fig. 4c). These experimental results confirmed that qOCT is a valid reference for compensation of signal attenuation by melanin in the RPE cells, and that qAF/qOCT reliably represents the amount of lipofuscin fluorophores in the RPE, independent of signal attenuations by RPE melanin and the pre-RPE media.

To further validate the quantitative relationship between qAF/qOCT and the A2E content, we imaged three pigmented LE rats (4 month old, 4 eyes) and three age-matched albino SD rats (4 eyes) using a commercially available PMMA slide with DAPI fluorescent dye (Fluor-Ref, Microscopy Education) as the reference target. Consistent with the results shown in Fig. 4, the compensated FAF intensities qAF/qOCT are higher in the 4 eyes of the LE rats than that in the 4 eyes of the SD rats (Fig. 5a). The A2E contents measured by LC/MS are also higher in the pigmented rats (Fig. 5b). Linear regression shows a linear relationship between qAF/qOCT and A2E content measured by LC/MS (R^2^ = 0.95, Fig. 5c).

The slope of the fitted line for qAF/qOCT vs A2E content measured by LC/MS shown in Figs. 4c and 5c can be defined as a calibration factor (*K*), which is determined by the quantum yield, molar extinction coefficient, and concentration of the particular fluorescent dye in the reference target, as well as the detection efficiency of the imaging system. The ratio between *K* of two different fluorescent dyes in the reference target, such as DAPI and A2E, can be calculated as:

1 $$\frac{{K}_{DAPI}}{{K}_{A2E}}=\frac{{Q}_{A2E}{C}_{A2E}{\varepsilon }_{A2E}{\eta }_{A2E}}{{Q}_{DAPI}{C}_{DAPI}{\varepsilon }_{DAPI}{\eta }_{DAPI}},$$

where Q, ε, C, and η are the quantum yield, the extinction coefficient, concentration of the fluorescent dye in the reference target, and the detection efficiency of the imaging system. The subscripts A2E and DAPI specify the corresponding reference target. According to Beer’s law, $Abs=\varepsilon Cd$, where *Abs* is the absorbance and *d* is the thickness of the reference target. To calculate the ratio *K*_*DAPI*_/*K*_*A2E*_, we used a spectrophotometer to measure the absorbance of each target in the band of the illumination light (465–495 nm)^31^. Since the detection efficiency of the imaging system for the two reference targets is the same, we have

2 $$\frac{{K}_{DAPI}}{{K}_{A2E}}=\frac{{Q}_{A2E}{\int }_{465}^{495}Ab{s}_{A2E}d\lambda }{{Q}_{DAPI}{\int }_{465}^{495}Ab{s}_{DAPI}d\lambda }=2.88.$$

In the calculation, ${\int }_{465}^{495}Ab{s}_{A2E}d\lambda$ and ${\int }_{465}^{495}Ab{s}_{DAPI}d\lambda$ are measured to be 51.35 and 1.58, the quantum yields of 0.003 for synthesized A2E and 0.04 for DAPI^39,40^ were used.

The *K*_*DAPI*_/*K*_*A2E*_ ratio, calculated by using the slopes of the fitted lines shown in Figs. 4c and 5c, is 3.04, close to the theoretical calculation shown in Eq. 2 with a difference of 5.5%. These results verified our theoretical analysis.

## Discussion

We have developed a VIS-OCT-FAF technology to quantitatively measure the true FAF intensities *in vivo* by compensating the signal attenuations caused by RPE melanin and the pre-RPE media. It is based on the idea that when the FAF and OCT images are generated simultaneously by the same light source, the depth-resolved OCT signals from the RPE undergo the same signal attenuations. Thus the OCT signals from the RPE can serve as an attenuation reference, and the attenuation caused by the RPE melanin and the pre-RPE-media can be removed by normalizing the FAF intensities to the OCT signals of the RPE. This approach does not need to measure the attenuations directly, as they are not directly measurable^29,30^. The compensated FAF intensities are free of attenuation caused by RPE melanin, as demonstrated by the present work, and by the pre-RPE media, as shown in our previous studies^31,32^. The VIS-OCT-FAF technology, in combination with a reference fluorescent target with known fluorescence efficiency and reflection coefficient placed in the intermediate retinal imaging plane, could lead to standardized accurate measurement of FAF intensities.

We used albino SD rats as a melanin negative control in the present work. SD rats, like many other albino animals, carry a missense mutation in the *TYR* gene that encodes a key enzyme, tyrosinase, for melanin biosynthesis^41,42^, resulting in the lack of melanin and albinism. It is remarkable to see that the initially measured quantitative FAF intensities qAF of the pigmented LE rats were lower than that of the age-matched SD rats, whereas after normalization with qOCT, the compensated FAF intensities qAF/qOCT of the LE rats are actually higher than the albino rats (Figs. 4a and 5a). This is rather unexpected as we thought the albino rats, with no protection of melanin against light exposure, could have higher amounts of A2E and thus higher FAF intensities since light is believed to be essential to A2E formation. The higher qAF/qOCT of pigmented rats is confirmed by direct LC/MS measurement of A2E contents. The linear correlation between qAF/qOCT and A2E contents (Figs. 4 and 5) shows that qAF/qOCT measurements correspond to the actual amounts of A2E in the RPE cells. Thus, qAF/qOCT represents the accurate FAF intensities emitted by A2E and other fluorophores in the RPE lipofuscin free of the attenuating influence of melanin and the pre-RPE media.

The clinical significance of accurate FAF intensities free of signal attenuation by the RPE melanin lies on the fact that there is a large variability in the distribution of melanin in the RPE, and a large variability from individual to individual^18^. In addition, local RPE hypopigmentation and hyperpigmentation are found to occur in the early stages of AMD^20^. Since FAF intensities are significantly reduced by RPE melanin due to signal attenuation, as demonstrated by the present work, it is difficult to compare qAF from individual to individual, and qAF from the same individual at different time points without taking the signal attenuating effect of RPE melanin into consideration. The true FAF intensities free of the attenuating influence of RPE melanin and the pre-RPE media by the VIS-OCT-FAF technology could have significant impact on the clinical usefulness of FAF.

Our theoretical analysis shows that the slope of the linear regression of qAF/qOCT vs A2E content in the RPE measured by LC/MS, i.e. the calibration factor K, is determined by the molecular characteristics and concentration of the fluorescent dye in the reference target. This has been confirmed by two separate experiments using two reference fluorescent targets with different fluorescent dyes. Thus, when using different reference target to image FAF, the measured qAF/qOCT can be related to each other if the characteristics of each target are known, making data obtained using different reference targets comparable. In Figs. 4c and 5c, the linear regression showed a residual qAF/qOCT when A2E/PC is zero, indicating the presence of other fluorophores in the RPE lipofuscin^13^.

In summary, we have developed a novel VIS-OCT-FAF multimodal imaging technology to obtain true FAF intensities. It can effectively compensate for the attenuation effects of RPE melanin and pre-RPE-media by normalizing the FAF intensities to the simultaneously acquired OCT signals of the RPE. The present work is a significant step toward standardization of quantitative FAF for clinical application.

## Methods

### Imaging system

The multimodal VIS-OCT-FAF imaging system design is similar to that used in our previous publications^31,32^ except a single reference target was used for both fluorescence and reflection reference. The system (Fig. 1) consists of two single-mode optical fiber-based spectral-domain OCT (SD-OCT) in the NIR and VIS spectrum, respectively. The NIR-OCT was used for aligning the imaging subject and finding the field of interest. The VIS-OCT has a wavelength of 480 ± 15 nm. The NIR-OCT used a super luminescent diode (SLD, wavelength: 850 ± 35 nm) as the light source. The VIS-OCT used a supercontinuum laser (SC, SuperK Extreme EXB-6, NKT Photonics, Denmark), in which the center wavelength and bandwidth were selected by a variable band-pass filter. The outputs of the two light sources were delivered to the source arms of the two 3 dB fiber couplers for the OCT systems. In the corresponding sample arms, the VIS and NIR light beams were first collimated and then combined by using two dichroic mirrors (DM1: DMLP505, Thorlabs, and DM2: NT43-955, Edmund Optics). The light beams were scanned and delivered to the eye by the combination of a pair of galvanometer scanners, a relay lens (L1, f = 75 mm, achromatic) focusing light on the intermediate retinal plane, and an ocular lens (L2, Volk lens, 60D). At the bottom of the field of view on the intermediate retinal-imaging plane, a PMMA reference target for FAF and OCT containing synthesized A2E as fluorescent dye is inserted.

The VIS-OCT had two reference arms with different path length, which were split by a non-polarizing beam splitter. The reference arm with longer path length was used for retinal imaging while the other was for imaging the reference-target. A shutter controlled by the computer blocks the light in the short reference arm when the retina is scanned. At the end of each imaging section, the shutter was opened to image the reference target by the VIS-OCT. The NIR- and VIS-OCT signals were detected by two spectrometers with the same parameters described previously^31,32^.

The FAF generated by the RPE lipofuscin was detected by a PMT module (H10723-20, Hamamatsu) with a 25 µm diameter pinhole. The fluorescence light was focused onto the pinhole by an f = 30 mm achromatic doublet after passing through the two dichroic mirrors, two long-pass filters (FGL515M, cut-on wavelength: 515 nm, Thorlabs) and a short-pass filter (FESH0750, Cut-Off Wavelength: 750 nm, Thorlabs).

### Image acquisition

The NIR-OCT real-time display was used for alignment before image acquisition. All the imaged retinas were at the same axial location on the OCT display and the optic disc was placed at the center of the raster-scan window. The NIR light was then turned off and visible light was turned on to capture VIS-OCT-FAF images. The right and left eyes of each rat were imaged four times. Each time, the alignment of the retina was slightly changed to test the repeatability of the results. All four images were later processed, and the average was used for quantification.

### *In vivo* imaging

We imaged both eyes of age-matched SD and LE rats. The experiments were conducted in agreement with the ARVO (the Association for Research of Vision and Ophthalmology) Statement for the Use of Animals in Ophthalmic and Vision Research and with the guidelines of the Florida International University’s Institutional Animal Care and Use Committee.

For imaging, an animal was anesthetized by intraperitoneal injection of ketamin (54 mg/kg body weight) and xylazine (6 mg/kg body weight). The eye to be imaged was treated with topical proparacaine hydrochloride ophthalmic solution (Akorn, 0.5%, USP) for topical anesthesia and tropicamide ophthalmic solution (Akorn, 0.5%, USP) for pupil dilation. A hard contact lens was put on the eye to prevent corneal dehydration and opacification. The sedated rat was restrained in an animal mount with five degrees of freedom.

### Image processing and calculation

The mathematical model for quantitative imaging of lipofuscin with an A2E-PMMA reference target can be expressed as:

3 $$\begin{array}{rcl}qAF & = & \frac{{I}_{FAF}}{{I}_{ref-AF}}=\frac{{I}_{0}{\tau }_{pre}^{2}(\lambda ){[1-{\rho }_{pre}(\lambda )]}^{2}{\xi }_{RPE}\,{A}_{d}\frac{\pi }{4}{\alpha }^{2}}{{I}_{0}{\xi }_{ref-A2E}\,{A}_{d}\frac{\pi }{4}{\alpha {\prime} }^{2}}\\  & = & \frac{{\tau }_{pre}^{2}(\lambda ){[1-{\rho }_{pre}(\lambda )]}^{2}{\xi }_{RPE}\frac{\pi }{4}{\alpha }^{2}}{{\xi }_{ref-A2E}\frac{\pi }{4}\alpha {\text{'}}^{2}}\end{array}$$

4 $$qOCT=\frac{{R}_{RPE-OCT}}{{R}_{ref-OCT}}=\frac{{I}_{0}{\tau }_{pre}^{2}(\lambda ){[1-{\rho }_{pre}(\lambda )]}^{2}{\rho }_{RPE}(\lambda )\frac{\pi }{4}{\alpha }^{2}}{{I}_{0}{\rho }_{ref-A2E}(\lambda )\frac{\pi }{4}\alpha {\text{'}}^{2}}=\frac{{\tau }_{pre}^{2}(\lambda ){[1-{\rho }_{pre}(\lambda )]}^{2}{\rho }_{RPE}(\lambda )\frac{\pi }{4}{\alpha }^{2}}{{\rho }_{ref-A2E}(\lambda )\frac{\pi }{4}\alpha {\text{'}}^{2}}$$

where qAF and qOCT represent the quantified FAF and OCT signals of the RPE. I_FAF_ and I_ref-AF_ are the fluorescent intensity of the fundus and the A2E reference target. R_RPE-OCT_ and R_ref-OCT_ are OCT reflectance signals of the RPE and Reference target, respectively. I_0_ is the intensity of the incident light and *A*_*d*_ represents detection sensitivity of the PMT. $\frac{\pi }{4}{\alpha }^{2}$ and $\frac{\pi }{4}{\alpha {\prime} }^{2}$ are solid angles comprising the light from the retina and reference target to the detector, respectively. ${\tau }_{pre}$, ${\rho }_{pre}$, and ${\rho }_{ref-A2E}$ are transmittance of the pre-RPE media, reflectance of the pre-RPE media, and reflectance of the A2E reference target, respectively. ${\rho }_{RPE}$ is the reflectance of the RPE layer. ${\xi }_{RPE}$ and ${\xi }_{ref-A2E}$ are the fluorescence efficiency of RPE lipofuscin and A2E in the reference, ${\xi }_{RPE}={C}_{L}{Q}_{L}{\varepsilon }_{L}{d}_{RPE}$, ${\xi }_{ref-A2E}={C}_{A2E}{Q}_{A2E}{\varepsilon }_{A2E}{d}_{ref-A2E}$, where C, Q, ε, and d are concentration, quantum yield, extinction coefficient, and the effective thickness of the fluorescent sample, respectively. The subscript L represents RPE lipofuscin. We then have:

5 $$\frac{qAF}{qOCT}=\frac{{C}_{L}{Q}_{L}{\varepsilon }_{L}{d}_{RPE}/{C}_{A2E}{Q}_{A2E}{\varepsilon }_{A2E}{d}_{ref-A2E}}{{\rho }_{RPE}/{\rho }_{ref-A2E}}$$

The equation can be further expressed as:

6 $$\frac{qAF}{qOCT}={K}_{A2E}\times \frac{{C}_{L}{Q}_{L}{\varepsilon }_{L}{d}_{RPE}}{{\rho }_{RPE}}$$

where ${K}_{A2E}$ is the calibration factor related to the fluorescent dye A2E in the reference target.

### RPE-choroid preparation and lipid extraction

Animals were euthanized immediately after imaging, and eyes were collected. The anterior segment of an eye was removed and the retina was carefully detached and discarded. The retina was carefully dissected to obtain the RPE-choroid preparation. Total lipids were extracted from the samples by a modified Bligh and Dyer method^43,44^. Briefly, the RPE-choroid preparation of each eye was mixed in 100 µl of H_2_O with a Bullet Blender (Next Advance, Troy, NY) at setting 7 for 3 min. Methanol (100 µl) was added to the sample and mixed, followed by adding 100 µl of chloroform (CHCl_3_) and mixing. The mixture was centrifuged at 14,000 rpm for 10 min in a tabletop microcentrifuge, and the lipid-containing lower phase was transferred to a collection tube. Extraction was repeated 4 times with 100 µl fresh chloroform added each time. Collected lipids in chloroform from each eye were pooled and dried in a SpeedVac (Savant Instruments, Holbrook, NY), flushed with argon, and stored at −20 °C in the dark until use.

### Quantitation of A2E by LC/MS

The amount of A2E in each sample was measured by reverse-phase LC/MS using a Shimadzu LC system (with a solvent degasser, two LC-10A pumps, and an SCL-10A system controller) coupled to a Triple TOF5600 mass spectrometer (Sciex, Framingham, MA), as described previously^45^. The flow rate of LC was 200 μl/min on a Zorbax SB-C8 reversed-phase column (5 μm, 2.1 × 50 mm, Agilent, Palo Alto, CA) with a linear gradient of mobile phase A (100%, methanol/acetonitrile/aqueous 1 mM ammonium acetate, 60/20/20, v/v/v, held isocratically for 2 min), then mobile phase B (100% ethanol with 1 mM ammonium acetate) by linearly increasing to 100% mobile phase B over 14 min then held for 4 min. The LC eluent was then delivered to the mass spectrometer ESI source. Instrument settings for positive ion ESI/MS and MS/MS analysis are as follows: Ion spray voltage (IS) = +5500 V; curtain gas (CUR) = 20 psi; ion source gas 1 (GS1) = 20 psi; de-clustering potential (DP) = +50 V; focusing potential (FP) = +150 V. The Analyst TF1.5 software (Sciex, Framingham, MA) was used for data acquisition and analysis. The measured A2E content in each sample was normalized with the amount of phosphatidylcholine 34:1 (PC 34:1), a major lipid of the retina^38^.
